# Vividness of Visual Imagery and Incidental Recall of Verbal Cues, When Phenomenological Availability Reflects Long-Term Memory Accessibility

**DOI:** 10.3389/fpsyg.2013.00001

**Published:** 2013-02-04

**Authors:** Amedeo D’Angiulli, Matthew Runge, Andrew Faulkner, Jila Zakizadeh, Aldrich Chan, Selvana Morcos

**Affiliations:** ^1^Neuroscience of Imagery, Cognition, and Emotion Research Lab, Carleton UniversityOttawa, ON, Canada

**Keywords:** episodic memory, incidental recall, multi-trace theory, visual imagery, vividness, VVIQ

## Abstract

The relationship between vivid visual mental images and unexpected recall (*incidental recall*) was replicated, refined, and extended. In Experiment 1, participants were asked to generate mental images from imagery-evoking verbal cues (controlled on several verbal properties) and then, on a trial-by-trial basis, rate the vividness of their images; 30 min later, participants were surprised with a task requiring free recall of the cues. Higher vividness ratings predicted better incidental recall of the cues than individual differences (whose effect was modest). Distributional analysis of image latencies through ex-Gaussian modeling showed an inverse relation between vividness and latency. However, recall was unrelated to image latency. The follow-up Experiment 2 showed that the processes underlying trial-by-trial vividness ratings are unrelated to the Vividness of Visual Imagery Questionnaire (VVIQ), as further supported by a meta-analysis of a randomly selected sample of relevant literature. The present findings suggest that vividness may act as an index of availability of long-term sensory traces, playing a non-epiphenomenal role in facilitating the access of those memories.

## Introduction

People often report they experience vivid spontaneous visual mental images in situations in which they have to recall something they did not expect to recall (*incidental recall*). Early imagery studies revealed that the spontaneous and involuntary appearance of a vivid visual mental image consistently occurred in response to certain memory conditions and tasks involving incidental recall. For example, upon asking subjects to remember the type of breakfast one had in the morning (Galton, 1880), the number of windows in one’s house (Shepard, 1966) or to verify a property of an experienced event with no aid of a current percept (Goldenberg et al., 1992) individuals often report vivid images. In such context, *vividness* is traditionally defined as a construct expressing the self-rated degree of richness, amount of detail (resolution), and clarity of a mental image, as compared to the experience of actual seeing (D’Angiulli and Reeves, 2007). Although vividness correlates with performance on certain memory tasks (Baddeley and Andrade, 2000), with arousal level (Barrowcliff et al., 2004; Bywaters et al., 2004), with positive emotional valence toward a stimulus (Alter and Balcetis, 2010), and with increased visual cortex activity (Farah and Peronnet, 1989; Farah et al., 1989; Sparing et al., 2002; Cui et al., 2007; Cattaneo et al., 2011, 2012), any attempt to clarify its function and its relationship to underlying processes still presents numerous challenges.

Manipulating vividness directly is difficult, and the lack of converging analyses has generally led to the use of correlational approaches that examine vividness predominantly as an index of individual differences in the ability to generate mental images. Furthermore, many preceding studies either confounded vividness with other variables, or did not appropriately interpret the validity criteria by anchoring the vividness construct to models of memory and verbal report underlying processes. This is a situation analogous to the one denounced years ago by Ericsson and Simon (1980) in the context of models of verbal reports, instruments such as vividness ratings/scale/questionnaires seem to be used in a brute empirical fashion, without considering a satisfactory *a priori* theory of the processes involved in the measurement instruments themselves. For the latter reason, it has been argued that there has also been confusion between issues of validity (e.g., discriminant or construct) and issues of reliability (e.g., specificity and precision). In the context of these challenges, the measurement of vividness has been hotly debated. As Pearson (1995) points out, vividness is usually measured using the Vividness of Visual Imagery Questionnaire (VVIQ) or its updated version, the VVIQ2 (Marks, 1995). However, these are not ideal measures for the experimental study of vividness *per se*, as they only measure the overall individual’s ability to generate vivid mental images (“trait vividness”), not differences between single experiences of mental imagery (“state vividness”). To study specific processes behind the phenomenon of vividness itself, it is more appropriate to use *trial-by-trial* self-reports in which the vividness of each individual mental image is rated immediately after its generation by the subject (Begg, 1988; Hertzog and Dunlosky, 2006; D’Angiulli, 2009; Pearson et al., 2011). The self reports were successfully employed in several previous studies, where the findings were consistent with both VVIQ research and new results outside the VVIQ’s realm of individual differences, which demonstrates that it is a reasonably robust measure (D’Angiulli, 2002, 2008, 2009; D’Angiulli, 2002; D’Angiulli and Reeves 2007; Alter and Balcetis, 2010; Rabin et al., 2010; Pearson et al., 2011). Despite these successes, so far there has been no clear empirical evidence showing exactly why trial-by-trial vividness reports should be considered more informative and reliable than the VVIQ. Do these sets of verbal reports reflect different or overlapping processes?

Many of the mentioned challenges could be mitigated by developing a model of processes underlying trial-by-trial vividness self-reports in visual mental image generation tasks, as opposed to just VVIQ measurement. One of the goals of the model should be to clarify the non-epiphenomenal role of the subjective vividness experience, a fundamental and difficult issue that continues to elude research efforts. An opportunity to gain some upper hand may be offered by conditions in which vivid imagery influences incidental recall in example situations such as the one mentioned earlier. The link between vividness and incidental recall was first suggested long ago (Richardson, 1969; Paivio, 1971) but the best evidence comes from studies showing that self-reported vividness is related with incidental recall of imagery-evoking verbal cues (Sheehan and Neisser, 1969; Sheehan, 1971, 1972b, 1973). In a typical paradigm devised by Sheehan (1972a), “vivid imagers” and “non-vivd imagers,” as defined by the VVIQ, were either intentionally or incidentally instructed to recall concrete (high imagery-evoking) and abstract (low imagery-evoking) words. Results showed that vivid imagers recalled concrete words significantly better in the incidental than in the intentional recall condition; whereas recall of abstract words was similarly poor in both conditions.

In another line of research, Neisser and Kerr used objective methods of mnemonic effectiveness and response time to study the spatial properties of visual imagery (Neisser and Kerr, 1973; Keenan and Moore, 1979; Kerr and Neisser, 1983). They asked the subjects to construct images in three different conditions according to presented sentences describing two objects in a given reciprocal spatial relation (concealed, next to/“pictorial,” far from/“separate”) and measured incidental recall rates of target verbal cues. Visual images acted as mnemonics in the concealed condition as well as the “pictorial” condition. If the procedure changed subtly and intentional learning was used instead, the objects in the concealed condition were recalled no better than the separate condition. The data from these experiments also showed that concealed images were less vivid than pictorial images, and response time was longer for less vivid images. Although instruction for imagery/recall had an effect on imagery vividness, incidental recall was invariably found to predict vividness even in studies that attempted to falsify Neisser and Kerr’s findings (Keenan, 1983).

The association between vividness and incidental recall is a relatively consistent finding across several different conditions and manipulations, and suggests that incidental recall could be used as the benchmark variable against which alternative hypotheses on the nature of imagery vividness and its function could be compared. Because older research had several shortcomings, Experiment 1 was designed to replicate, generalize, and extend said relationship. Most of those studies used global or delayed self-report of vividness. In addition, image generation time was confounded with vividness, and most paradigms did not clearly show whether the observed effects were discriminatively and specifically linked to recall processes (refer to Sheehan, 1973, for one exception). Furthermore, individual differences were often globally defined by the VVIQ, such that “good” versus “poor” imagers determined “high” versus “low” vividness, respectively. Finally, the lack of control for factors relating to the cued words themselves was a consistent problem in previous research. In the present research, a direct imagery and incidental recall paradigm were used, and several verbal properties were controlled for (age of word acquisition, word familiarity/frequency, imageability, and concreteness).

We compared two hypothetical cognitive components of mental image generation from verbal descriptions, which possibly could account for the outcomes of Experiment 1. If the relationship between vividness and unexpected recall were contingent upon shared processing while encoding the cues in the study phase (image generation), a possible relationship may be explained by *depth of elaboration* (Craik and Lockhart, 1972; Eysenck and Eysenck, 1980). The more time spent elaborating the imagined material, the more subjectively vivid the material should be. Subsequently, this should lead to better retention and recall in the test phase (free incidental recall). The main predictions derived from this hypothesis were that: (1) a direct relationship between image latency and incidental recall should exist, as should a relationship between incidental recall and self-rated vividness; (2) however, the correlation between vividness and incidental recall should be accounted for by image latency. Therefore, the correlation between vividness and incidental recall should be non-significant and/or correspond to a small effect size when image latency would be controlled for.

A possible alternative based on neurocognitive considerations is that vividness ratings rely on an index of the availability of multiple sensory traces in long-term memory (Hintzman and Block, 1971). Thus, because the strength of vividness would reflect the magnitude of the networks of sensory traces consolidated from episodic memory (Morris and Hampson, 1983; Rabbitt and Winthorpe, 1988), higher vividness ratings should be associated with better incidental recall performance (higher likelihood of accessing long-term traces). This model would also predict that the relationship between vividness and incidental recall can be partly explained by individual differences in participants’ ability to access long-term memory sensory information based on the prior estimate of availability supported by vividness judgments. The latter aspect could be conceived as a “meta-imagery” contribution, where the vividness judgment may reflect “a judgment of the richness of the current image combined with an estimate of the additional sensory information that could be incorporated, should the task requirements change.” (Baddeley and Andrade, 2000; p. 141). Consequently, individuals with greater metacognitive ability should experience more vivid images, be more efficient and faster in generating images, and yield higher incidental recall than the individuals who possess a reduced metacognitive ability. If greater vividness were related to greater incidental recall accuracy, and the relationship was not simply due to longer image latencies, then this would support the hypothesis that vividness acts as an index of stored memory trace availability, and plays a non-epiphenomenal role in determining the likelihood of accessing such memories in long-term memory.

In all the following experiments, explicit instructions to generate mental images was adopted as this manipulation has proven to be perhaps the most reliable and most direct way to ensure that participants are actually generating mental images, as shown by converging evidence from hundreds of studies showing that the report of having an image at request is associated with behavioral, neural, or clinical neuropsychological indices. In addition, while direct interference of imagery on low-level perception is an established phenomenon (Craver-Lemley and Reeves, 1987), the opposite effect, direct interference of low-level perception on imagery, is either weak and ubiquitous (see D’Angiulli, 2002) or is based again on introspective reports (as in Baddeley and Andrade, 2000). Therefore, the latter manipulations are no better or different than the ones we used for verifying the employment of imagery.

## Experiment 1

### Method and materials

#### Participants

Participants were 26 first-year university students *age range* = 17–25; 14 female and 12 male). None had participated in an imagery study before (Campos et al., 2007). Participants signed up through a subject pool within 3 weeks of beginning introductory psychology courses, with 2% credit toward their final grade used as incentive. No significance was found for gender or age against any factors, so these variables were dropped from further consideration.

#### Stimuli

A body of 60 verbal description-cues from previous research (D’Angiulli, 2002:1]; available in D’Angiulli, 2001a) were matched with regards to noun or compound word frequency, imageability, concreteness, and reading time. These cues included single-noun and double-noun descriptions comprising both animate (e.g., dog, cat) and inanimate objects (e.g., car, bottle). The present data showed no significant differences between the two subsets of stimuli in terms of vividness or latency of elicited imagery. Secondary analyses indicated that these descriptions were rated as emotionally neutral, with negligible inter-item variability along a simple emotional rating scale (D’Angiulli, 2001b). In addition, the 10 noun-cues were selected from earlier research (Paivio et al., 1968) to use as buffer items during the incidental recall phase of the experiment (i.e., to filter out recency and primacy effects during recall). The 60 cues were presented in random order, preceded by five buffer noun-cues and followed by five other buffer noun-cues (which were presented in a fixed order).

Stimuli properties previously shown to intercorrelate were controlled for. Verbal cues with higher concreteness levels were shown to be recalled at significantly higher rates (Paivio, 1971), as were high frequency words (e.g., Miller and Roodenrys, 2009). Imageability, which refers to how easily a mental image can be generated from a word, has been correlated with concreteness (Tse and Altarriba, 2007). Age of acquisition, which refers to the average age a word enters a subject’s lexicon was indirectly controlled for, as it is highly correlated with both imageability (Ma et al., 2009) and concreteness (Barry and Gerhand, 2003). The well-validated MRC Psycholinguistic Database (Clark, 1997) was used to ensure the words used for cuing had approximately the same scores on these factors. Because it was assumed that vividness is an image-specific process, and it could not be rated if an image does not reach to conscious awareness, all cases rated “no image” were eliminated from our analysis.

### Procedure

The protocol for Experiment 1 was approved by the Carleton University Research Ethics Board.

#### Image generation phase

Participants were seated facing a computer monitor and pressed the right mouse button to begin each trial. Upon clicking the mouse, an alerting beep was sounded, followed 250 ms later by the display of a noun-cue at the center of the screen. Participants were instructed to read the cue silently and as quickly as possible. They were immediately asked to generate an image that corresponded to the noun-cue. Participants were required to press the right mouse button again when they considered their image to be complete, and at its most vivid.

Upon pressing the button, another alerting beep was sounded, followed 250 ms later by a horizontal array of seven choices appearing near the bottom of the screen. From left to right, each button was labeled with one of seven vividness level descriptions in a seven-point scale format [(1), “no image”; (2), “very vague/dim”; (3), “vague/dim”; (4), “not vivid”; (5), “moderately vivid”; (6), “very vivid”; and (7), “perfectly vivid”], as in Marks (1995. Time was taken to familiarize participants with the rating system during pre-test practice sessions. Participants used the mouse to click on one of these seven buttons, and were instructed to rate any failure to generate an image as a “no image.” There was no deadline for their response.

Following the vividness response, the array of buttons disappeared and the display reverted back to a screen instructing the participant to click the mouse when they were ready to begin the next trial. In an effort to minimize imagery persistence between trials, stimuli were presented in random order with a minimum inter-trial interval of 5 s (Craver-Lemley and Reeves, 1987). Participants were not informed that latency times were covertly measured. Button presses were justified as a means to signal a complete image, which was ready to be rated, and prompt the appearance of the vividness scale buttons.

#### Free incidental recall phase

After completing the image generation phase, participants took a 20 min break. Afterward, they were asked to return to the lab to fill out additional paperwork, to receive course credit, and complete the debriefing process. Prior to the image generation phase, participants had not been informed that they would be required to recall any of the stimuli. Upon their return, precisely 30 min from the end of the image generation phase, they were asked to complete the incidental recall task, wherein they were required to recall and record as many of the previously read descriptions as possible.

Each phase of the experiment was exclusively conducted by one of two paid undergraduate research assistants. Both research assistants received training in their module, yet remained naïve to the purposes and hypotheses of the study. Final debriefing was conducted through an exit interview with the principal investigator.

### Results and discussion

Preliminary analyses were conducted on the empirical distributions of raw response times (RTs) for each level of vividness (except level 1 = “no image”). A total of 1490 valid observations were available after all cases with a rating of “no image” (5% of total trials) were removed. Data were binned using the smallest increment that did not make the histograms appear too irregular. From the initial binning it became apparent that our RT data could be fitted by an ex-Gaussian – that is, the convolution of an exponential with a Gaussian. This ex-Gaussian model has been used successfully in several experimental paradigms (for reviews, see Ratcliff, 1979, 1993; McNicol and Stewart, 1980; Luce, 1986) to fit explicit theoretical distribution functions and to give convenient summary of empirical RT distributions. The assumption of the ex-Gaussian model is that RT is the sum of two other random variables, one distributed as a Gaussian and one distributed as an exponential (Luce, 1986). Previous work (D’Angiulli and Reeves, 2002) has supported the hypothesis that the ex-Gaussian model reflects the time to retrieve images from memory so that “image generation” can be essentially reduced to “retrieving images from memory.” Therefore, variations in each of ex-Gaussian parameters across vividness levels could be assumed to describe the core underlying generative processes common to both imagery and incidental recall. The ex-Gaussian model was fitted using a robust regression method due to Hoaglin et al. (1983).

To ensure the ex-Gaussian reflected the shape of the group data, and the shape of the individual data, the model was first vincentized for individual data, and then averaged over vividness levels. Histograms were constructed by pooling the raw RTs from each vividness level over subjects, irrespective of the individual source of the RTs. This method has been used in situations where there are too few trials for single subjects (see Ratcliff, 1979). We verified whether the related observations were serially independent and not autocorrelated for each subject, if so we could assume independence of collective observations (see Neter et al., 1996). In our case, the Durbin–Watson autocorrelation test statistic *D* clearly exceeded the upper bound in the assessment of each subject [*d_u_* > 1.62; α = 0.05; *n* = 60; lag = 1] as well as for each vividness level submitted to fitting, thereby showing no autocorrelation.

Table 1 shows the ex-Gaussian fit to the distribution histograms of RTs obtained for each vividness level. For each distribution, the ex-Gaussian fit explained at least 68% of the variance associated with RTs. The general distribution of the vividness data showed the median rating was a value of 4 (“non-vivid”). Examination of each vividness level regressed onto RTs showed both distributions were best summarized by piecewise linear regressions of opposite slope. These data supported a clear split between *vivid* (rating values 5–7) and *non-vivid* (2–4) observations.

**Table 1:** **Results of the ex-Gaussian fit to empirical image latency distributions in unconstrained image generation phase of Experiment 1 (see text for details)**.

Vividness	λ	μ	*r*^2^	MRT	SDRT	*N*
2	18046.0	1500.0	0.68	19547.2	26055.7	17
3	8807.0	2500.0	0.95	11307.2	14520.5	31
4	18641.0	5000.0	0.95	23641.2	28188.5	64
5	12027.0	2500.0	0.96	14527.3	16280.8	174
6	11612.0	2500.0	0.96	14112.3	15416.4	328
7	8162.0	5000.0	0.99	12162.0	18938.2	449

*All values reported in the table – except the ones corresponding to n’s – are in ms; 500 < σ < 1000. Robust regression with Ramsay’s weighting function*.

**Figure 1:**
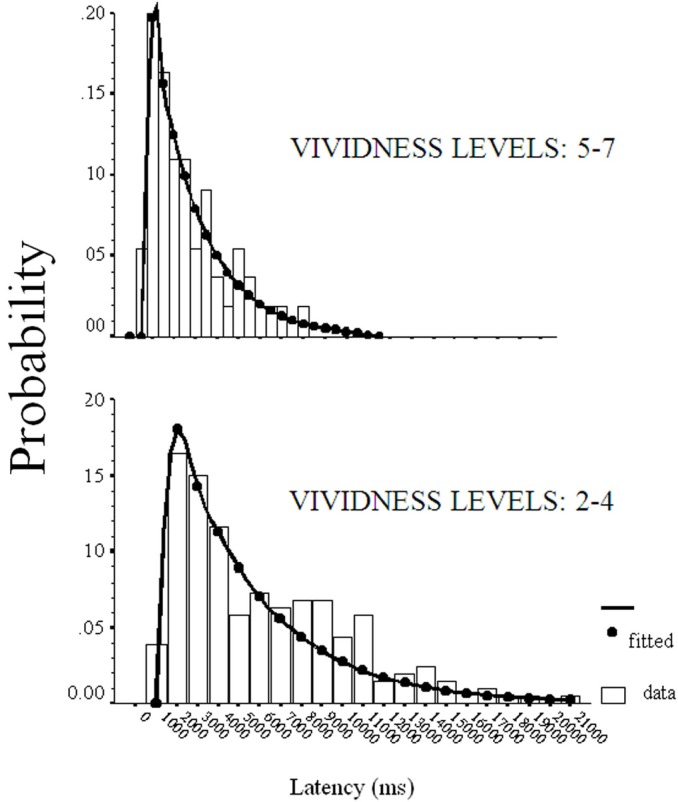
**Ex-Gaussian model-fit of RT distributions for images rated with vividness 2–4 and 5–7 in Experiment 1**.

The Gaussian of both vivid images (levels 5–7) and less vivid images (levels < 4) are reported in Figure 1. Both distributions have comparable standard deviation, as evidenced by the left tail of the distributions. However, the distribution of less vivid images is delayed >500 ms, as evidenced by the shift on the time axis. Consistent with previous findings (D’Angiulli and Reeves, 2002), more vivid images were typically associated with shorter Gaussian latency components than were less vivid images. It is important to point out the enormous variability in the response latencies, and that the relationship between vividness could not be easily guessed by naïve participants. Therefore, it is rather implausible that the observed pattern might be due to response-bias based on an explicit or conscious criterion-shift, or set of decisions, since this would have required the participants to first tacitly simulate the ex-Gaussian model, and then retrofit their responses coherently to the model to produce the observed pattern. Because this would have to be done uniformly by all participants, the variability should have been much more contained than what we observed.

**Figure 2:**
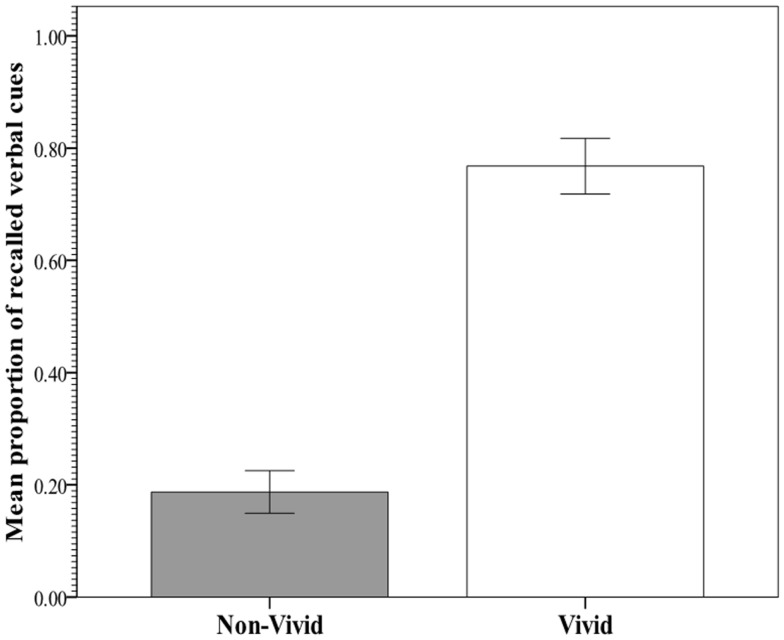
**Mean proportion of incidental recall for verbal cues corresponding to vivid (rating: 5–7) and non-vivid (rating: 2–4) images in Experiment 1**.

**Figure 3:**
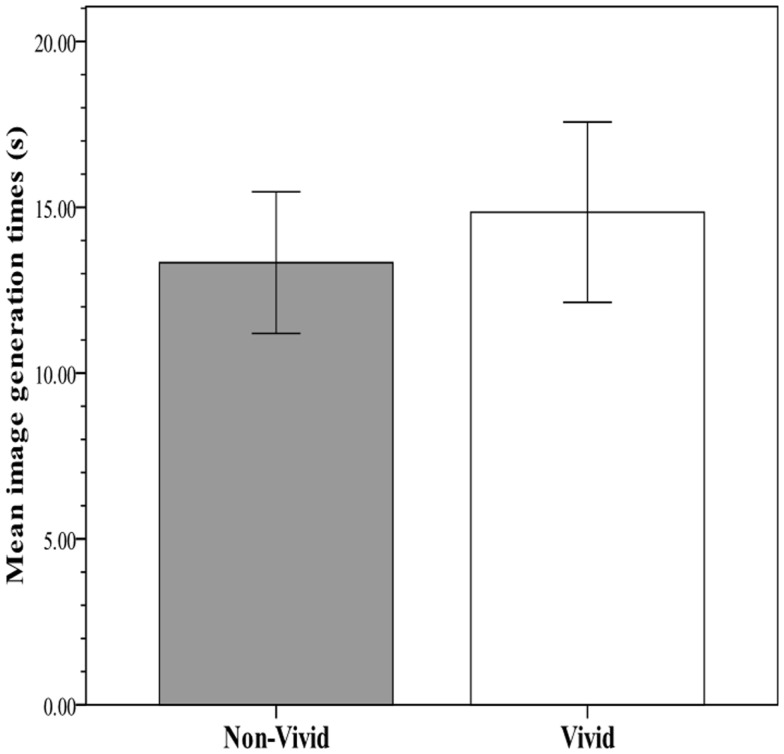
**Mean image generation time for verbal cues corresponding to vivid (rating: 5–7) and non-vivid (rating: 2–4) images in Experiment 1**.

The key analysis examined the predictability of recall and RTs from vividness rating category (non-vivid versus vivid). In an effort to meet assumptions for parametric procedures and augment robustness to violations, the distribution of RTs was normalized with a logarithmic transformation, after which no multivariate outliers were detected. Figure 2 shows the within-subject mean proportion of incidentally recalled imagery-evoking verbal cues presented during the image generation phase against the rated vividness level. Figure 3 shows the within-subjects mean RTs of image generation against the rated vividness level (for presentation, RT data are expressed as *seconds*, derived from antilog transformation). The proportion of recalled cues corresponding to vivid images was 0.77 (SE = 0.05), whereas the proportion of recalled cues corresponding to non-vivid images was 0.19 (SE = 0.04). A paired samples test showed the difference to be significant [*t*(25) = 6.69; *p* < 0.0001], explaining 74% of the variance. In contrast, the mean RTs for vivid (14.8 s, SE = 2.71) and non-vivid (13.33, SE = 2.14) cues did not differ [*t*(25) < 1, *p* = 0.34; *R*^2^ < 0.01].

A linear regression analysis examining the effect of individual differences on the total number of images recalled showed that 14% of the variance in incidental recall accuracy was explained by participants’ average vividness rating [*F*(1, 25) = 4.05, MS*_e_* = 0.38, *p* = 0.05]. Therefore, the role of individual differences was modest and its effect size (*r*) was significantly smaller than that of vividness described earlier (0.86 versus 0.37, *z* = 3.07, *p* = 0.002).

A two-predictor model (stimulus and vividness) was fitted to the data to test the hypothesis regarding relationship between vividness and recall. Stimulus was plotted as a nominal factor, in which each category was a noun-cue. It was included as a predictor to ensure vividness effects were not due to the tendency for some words to produce more vivid images than others. The resulting model [*Predicted logit of (Recall)* = 0.664 + β1^*Vividness^ + β2^*Stimuli^] was statistically reliable, χ^2^(62, 1441) = 340.969, *p* < 0.001 (see Appendix A for analysis details). According to the model, greater vividness ratings for noun-cues predicted recall with an overall success rate of 72.2%. The model correctly classified 83.7% of unrecalled cues and 54.7% of recalled cues. Stimulus and vividness generate a statistically significant predictive model for recall (see Appendix) that accounted for 28.3% of the variance in incidental recall. No change was observed if the model was fit to predict recall when response time was added as a predictor [χ*^2^*(65, 1490) = 389.437, *p* < 0.001]. RT did not exert an influence on the model (*B* = −0.002, *p* = 0.587), which further confirmed the null effect of RT on recall. Therefore, vividness could not account for recall accuracy simply because participants spent more time imagining the items corresponding to the verbal cues.

A linear mixed model was fit to the data to assess the contribution of stimulus and RTs to linear change in vividness of imagery. The variables in the model were evaluated by a Type III test. Since the sample size was not large, Restricted Iterative Generalized Least -squares (RIGLS) was used (Goldstein, 1986). Stimuli and RT had a significant effect on vividness [*F*(59, 1000) = 1.59, η^2^ = 0.086, *p* < 0.05], and *F*(1, 1103) = 5.17,η^2^ = 0.005, *p* < 0.05, respectively. Therefore, because the effects were small, RTs and stimuli influenced vividness only minimally. There was no interaction between stimuli and RTs (*F* < 1).

To determine if recall and vividness ratings were affected by the verbal properties of the word stimuli that were not kept constant during stimulus selection, correlation analyses were conducted on age of acquisition, and familiarity versus recall. No significant relationship was found between the percentage of participants that recalled a cue, and either age of acquisition (*r* = 0.213, *p* = 0.317) or familiarity scores (*r* = 0.118, *p* = 0.445). In addition, effects of stimuli regressed onto vividness, recall and RTs all explained less than 0.5% of the variance.

The results of Experiment 1 implicate vividness ratings as a predictor of incidental recall for imagery-evoking cues. The effect of individual differences in imaging ability on incidental recall was much smaller than the effect of vividness. Because image latency was unrelated to incidental recall, and inversely related to vividness, these data were incompatible with the depth of elaboration account. Because the effect of vividness on incidental recall for verbal cues was tested, the influence of expectancy and demand characteristics were minimized. These results support the validity of vividness as a measurable construct, and as an entity which may represent real underlying memory processes. Vividness ratings likely reflect a process which provides a natural mnemonic for unexpected retrieval of implicitly coded information (see Kosslyn et al., 2006).

## Experiment 2

Although Experiment 1 did not include a measure of VVIQ, incidental and intentional recall has traditionally shown a modest correlation with the VVIQ and VVIQ2, with average effect sizes generally of about *r* = 0.13 (see McKelvie, 1995; Dean and Morris, 2003). More recent evidence suggests the relationship between VVIQ2 and trial-by-trial vividness ratings is weak to moderate (*r* < 0.20) (D’Angiulli, 2001a; D’Angiulli and Reeves, 2007). Also, the patterns of results from Sheehan (1971, 1972b) suggest the quality of imagery is contingent upon properties of the stimuli within the setting of each trial, and predicts incidental free recall and recognition performance. Lastly, other studies found the modest correlation between trial-by-trial ratings and VVIQ holds only for female participants (Sheehan, 1971, 1973).

In contrast with these findings, Pearson et al. (2011) reported large predictive effects of both trial-by-trial vividness ratings and VVIQ2 scores when related to bias in reporting a dominant pattern during a binocular rivalry task. The underlying assumption was that similar metacognitive processes (i.e., knowing how and what the observer knows about his/her own processes of visual mental imagery) would be used in trial-by-trial vividness ratings and in VVIQ2. If this assumption is correct, the overlapping processes could shed some light on the results of our Experiment 1. One interpretation of the results of Experiment 1 is that trial-by-trial vividness ratings may be accounted for by the same metacognitive judgment processes involved in responding to the VVIQ2. Experiment 2 was designed to examine the putative relationship between vividness ratings and VVIQ2. If the association between the VVIQ2 and vividness ratings were confirmed in Experiment 2, then one may also explain the basis through which vividness ratings could predict incidental recall in terms of the overlapping metacognitive processes involved in the VVIQ2.

The design of Experiment 2 was a variation of the paradigm used by Baddeley and Andrade 2000). Upon completing the VVIQ2, female participants were asked to read a short description of a static or dynamic scene, and press a key upon generating complete visual mental image. Participants then rated the vividness and the subjectively perceived latency of the image on a trial-by-trial basis. If, as the results of Experiment 1 would suggest, vividness ratings are based on an index of multiple sensory traces available in long-term memory, this account would predict: (1) higher trial-by-trial vividness ratings for dynamic scenes than static scenes, and (2) a negative (i.e., inverse) relationship between trial-by-trial vividness and perceived imagery latency. The VVIQ2 should correlate with trial-by-trial vividness ratings from both dynamic and static scenes, but should not relate to perceived imagery latency when the effects of vividness are removed.

Conversely, if the VVIQ2 accounts for most of the relationship between trial-by-trial vividness ratings and perceived imagery latency, then vividness judgments could be attributed to similar individual metacognitive skill differences involved in the two types of vividness measures (Baddeley and Andrade, 2000; Pearson et al., 2011). However, because dynamic mental imagery capacitates working memory more than static mental imagery, fewer resources are available for concurrent metacognitive processes. Then, under such circumstances one would expect less vivid images for dynamic scenes than static ones.

### Methods and materials

#### Participants

Participants were 44 female undergraduate students (*age range*: 18–25). Participants signed up through a subject pool, with 2% credit toward their final grade used as incentive. All participants had normal or corrected-to-normal vision, and no reported or documented learning disabilities. Participation required the attendance of two appointments. The first appointment was a preliminary screening session, where participants filled out the VVIQ2 and individual data. The second appointment was the experimental session. Five potential participants were excluded from the experiment, as they were unable to evoke the images as required.

#### Materials

An adaptation of 17 static and 17 dynamic scene descriptions were used (Baddeley and Andrade, 2000; Experiment 4, see Appendix A, p. 144). The scenes were adapted such that words including British content (e.g., Big Ben) were substituted with equally long words describing North American content (e.g., CNN Tower) which were validated through pilot experiments. During the screening phase, a question from the visual portion of the *procedure for assessing expectations on the vividness of imagery* was asked (Baddeley and Andrade, 2000; see Appendix C, Q2, Question 2, p. 145). After the experimental phase, a tacit knowledge assessment procedure was administered.

### Procedure

The protocol for Experiment 2 was approved by the Carleton University Research Ethics Board.

Participants were given instructions, and 10 min of practice with five dynamic and five static imagery scenes. Between each practice trial, participants were required to report how well they could control each image. Only participant ratings with vividness greater than “extremely slow” (1) for 80% of the practice trials qualified for the entire experiment. One participant was eliminated from the initial pool under such criteria. Upon completing the practice session, participants verbally repeated the instructions to the experimenter to ensure the instructions were understood.

Participants were instructed to silently read a description of a dynamic or static scene displayed on a computer screen, which occurred 250 ms after an alerting beep. The experiment consisted of 17 dynamic, and 17 static descriptions. Participants were tested individually, and the procedure lasted approximately 40 min. Upon reading each description, participants were required to press a key to indicate the description was understood. Participants were instructed to imagine the description with their eyes open, and as seen from the front. Outline drawings were shown as examples before the experiment began. Each description was presented in random order with an inter-trial interval of 5 s (Craver-Lemley and Reeves, 1987). Upon forming a complete mental image, participants were required to press a button on a mouse. Four seconds after the button press, participants were shown buttons to rate perceived vividness, and perceived latency of the images. Participants were asked to rate their image as “complete” or “finished” when the image was maximally clear and detailed (see Cocude and Denis, 1988). Participants were required to rate their mental image as they had experienced it at the time of the key press. There was no deadline for the rating responses.

The presentation order of the scales was randomized, such that vividness could follow or precede perceived imagery rating. The second rating task followed immediately after the first rating response. The vividness scale consisted of a horizontal array of seven buttons appearing at the center of the screen. From left to right, each button was labeled with a short description corresponding to one of seven levels of the vividness scale used in Experiment 1. The imagery latency (speed) scale consisted of a horizontal array of seven buttons appearing at the center of the screen. From left to right, each button was labeled with a short description corresponding to one of the seven levels: from “extremely fast” (7), to “extremely slow” (1). Valid trials were defined by vividness greater than 1. Subjects were instructed to give a “1” response if they were unable to form a mental image. Upon completing the experiment, participants underwent a post-experimental interview, wherein they quickly described what they had imagined for seven randomly probed descriptions from both dynamic and static condition. Post-experimental interviews were concluded with the tacit knowledge assessment procedure (Baddeley and Andrade, 2000), and included the following question:

“We are interested in knowing if you think that there was a relationship between how vivid your images were and other factors. Please just tell us what you expect or think, please do not use images to answer the question, we are just interested in what you predict or think about things that may be related or may determine the vividness of your images.”

### Results and discussion

To eliminate effects of discrepant scales, total scores for the VVIQ2 were converted to mean vividness values through a simple linear transformation. The transformation resulted in a seven-point scale; henceforth, referred to as *mean vviq2*. As in Experiment 1, we considered only valid responses. The rate of excluded invalid trials was approximately 3% (level 1 = “no image”), a proportion similar to Experiment 1. On average, images were reported as moderately vivid, and were produced at a relatively fast perceived latency in both static (*M*_viv._ = 5.31, SD_viv._ = 0.55; *M*_speed_ = 5.40, SD_speed_ = 0.39) and dynamic (*M*_viv._ = 5.29, SD = 0.77; *M*_speed_ = 5.59, SD_speed_ = 0.62) conditions. Paired samples contrasts showed dynamic imagery was perceived as significantly faster than static imagery [*t*(38) = 2.52, *p* < 0.025]. However, mean vividness ratings did not differ between the two conditions [t(38) < 1, *p* = 0.797]. The latter result differed from Baddeley and Andrade’s findings (Experiment 4). (They found dynamic imagery was significantly less vivid than static imagery). Images produced for the VVIQ2 were significantly more vivid (*M*_vviq2_ = 5.68, SD_vviq2_ = 0.62) than vividness for static images [*t*(38) = 3.88, *p* < 0.0001], and dynamic images [*t*(38) = 2.83, *p* < 0.01].These data may be interpreted as evidence that participants were generally much more confident in their imagery abilities than what they were capable of demonstrating during the experimental procedure. The discrepancy between trial-by-trial vividness level and VVIQ2 imply a lack of agreement between metacognitive judgment as measured through the VVIQ2, and verbal reports specific to the actual imagery task.

Table 2 shows correlations among all measures. VVIQ2 was significantly correlated with vividness of static imagery, but was not related to vividness of dynamic imagery, nor perceived latency in both static and dynamic imagery conditions. A very strong inverse relationship between trial-by-trial vividness ratings and perceived imagery latency was observed in both static and dynamic imagery conditions, with strong to marginal evidence of the same trends in crossed conditions.

**Table 2:** **Correlation matrix among VVIQ2 and self-reported image vividness ratings and perceived generation speed in dynamic and static imagery conditions of Experiment 2**.

	Dyn. vividness	Stat. vividness	Dyn. speed	Stat. speed
VVIQ2	0.259	0.505**	0.044	−0.219
Dyn. vividness	–	0.679**	−0.626**	−0.652**
Stat. vividness	–	–	−0.282†	−0.531**
Dyn. speed	–	–	–	0.622**

Dyn., dynamic imagery condition; Stat., static imagery condition. *N* = 39. †*p* < 0.10, **p < 0.01.

**Figure 4:**
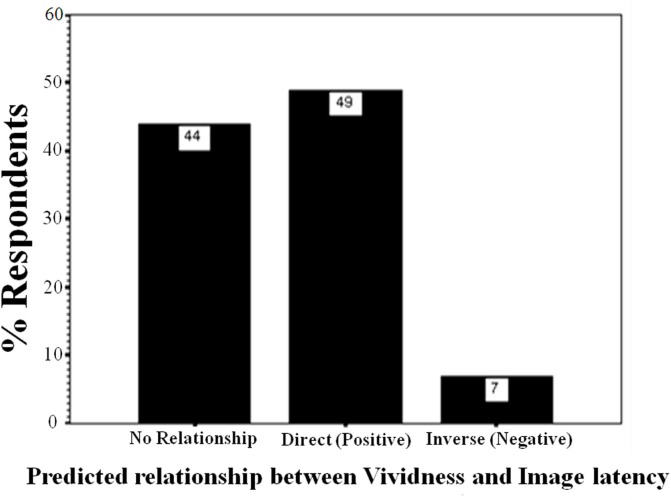
**Percentages of participants predicting what type of relationship they tacitly think there should be between vividness ratings and speed of imagery in Experiment 2**.

Whereas vividness ratings correlated with perceived latency, the VVIQ2 did not. These data provide very weak evidence validating the VVIQ2, when the criterion is a self-report, subjective third variable. Logically, one would not expect any predictive success of VVIQ2 in relation to a behavioral variable such as incidental recall. The observed patterns were analyzed to determine if they could be predicted by expectations or tacit knowledge (Pylyshyn, 2003). There was no significant difference in the number of participants expecting vivid imagery to be less or more vivid than static imagery (χ^2^ < 1). Figure 4 describes participant responses concerning self-rated predictions about the type of relationship they expect to exist between perceived vividness and perceived imagery latency, as documented during the preliminary screening session. Most participants predicted a positive relationship, or no relationship between vividness and imagery latency. One participant correctly predicted the inverse relationship. Upon removing the data of this participant from the analysis, there were no significant differences between results.

In conclusion, the association between the VVIQ2 and vividness ratings was not observed consistently in both the conditions of Experiment 2, and if collapsed across conditions (static and dynamic) the effect becomes modest and not significant. VVIQ2 also failed to validate against a third self-report criterion variable (perceived image latency). If the VVIQ2 assesses individual differences in metacognitive ability, it seems implausible that such abilities would predict incidental recall. Because trial-by-trial vividness predicted incidental recall, the metacognitive aspects assumed to be reflected by VVIQ2 do not appear to influence vividness and the mental imagery process to a significant degree.

### General discussion

Despite controlling for imageability, concreteness, age of acquisition, and verbal frequency/familiarity, the results from Experiment 1 showed a positive relationship between vividness ratings and incidental recall of imagery-evoking cues. These results are not consistent with depth of elaboration, as faster image generation latencies accompanied higher vividness ratings, a pattern opposite to what depth of elaboration would predict. Furthermore, because depth of elaboration predicts a positive correlation between incidental recall and image generation time, it again fails to account for the data from Experiment 1.

Our findings are compatible with an alternative model of vividness processes based on multi-trace memory theory (MMT; Moscovitch et al., 2005). This model proposes that vividness ratings are based on an index of the availability of multiple sensory traces in long-term memory, the strength of vividness reflecting the magnitude of the networks of sensory traces that have been consolidated from episodic memory. This is described by the inverse relationship between vividness ratings and image latency (the “vivid-is-fast” relation). Thus, higher vividness ratings are associated with higher likelihood of incidental recall, as shown by the data of Experiment 1.

The follow-up results observed in Experiment 2 showed that individual differences, as measured by the VVIQ2, are not a viable account for the relationship in Experiment 1 between vividness and incidental recall. Most important, the results of Experiment 2 also suggest that if there were metacognitive aspects involved in trial-by-trial vividness ratings, they would not likely be the same ones underlying VVIQ measures. Taken together the results of Experiment 1 and Experiment 2 are consistent with those observed in a meta-analysis we conducted, representing 5% of the literature pertaining to “vividness” and “VVIQ” (reported in Appendix B). The proportion of significant and non-significant experimental outcomes for trial-by-trial vividness ratings and VVIQ factor effects were calculated. For behavioral, cognitive, and neural measures, a greater number of significant experimental outcomes accompanied trial-by-trial vividness ratings than the VVIQ. Furthermore, the correlation between VVIQ scores and trial-by-trial vividness ratings for 21 entries showed an average correlation of 0.15, and variability in these values ranged from *r* = −0.27, to *r* = 0.64. Consistent with the results of experiment 2, these additional results support the contention that trial-by-trial vividness self-reports and VVIQ scores share some descriptive properties of visual imagery. However, trial-by-trial vividness ratings seem to resolve the construct of mental imagery with much greater reliability. Although metacognitive processes may be occurring in single trial judgment, it is perhaps more parsimonious to assume that vividness ratings are mostly a form of *Level 2 retrospective verbal reports* (Ericsson and Simon, 1993).

Considered as retrospective verbal reports, vividness ratings may be based on a direct translation of residual top-down sensory traces available in long-term memory (D’Angiulli and Reeves, 2002), wherein vividness intensity is proportional to the magnitude of sensory traces available. This statement agrees with a number of neurocognitive considerations borne out of MMT research. According to that theoretical framework, each sensory trace is distributed across the cortex, such that various distributive patterns are unique to a specific sensory input, and is distinct from all other distributive patterns (Hintzman, 1976). Sensory traces are thought to be indexed by the hippocampus (Ryan et al., 2001), and integrated into a mental image by the cuneus, precuneus, and occipital lobes (Svoboda et al., 2006; Svoboda and Levine, 2009; Cabeza and St. Jacques, 2007). However, hippocampal indexing becomes less influential as each individual sensory trace is integrated into cortical networks through successive (re)presentations (Takashima et al., 2009). Mental images are consolidated neural patterns that correspond to these “synthetic” sensory long-term traces, whose levels of interconnectedness are correlated to their perceived reportable vividness (Rabin et al., 2010).

Our study also indicates that although the VVIQ or VVIQ2 may very well measure an individual’s ability to generate vivid mental images (“trait vividness”), it likely lacks the resolution to measure an individual’s ability to experience vivid mental images in specific situational contexts (“state vividness”). To study specific processes behind the phenomenon of vividness itself (rather than “trait vividness”), it is perhaps more appropriate to use trial-by-trial self-reports, wherein vividness is rated immediately after its generation (Begg, 1988; Hertzog and Dunlosky, 2006; D’Angiulli, 2009; Pearson et al., 2011). Such self-reports have met with compounding success progressing beyond the VVIQ’s realm of individual differences, while remaining generally consistent with it. Vividness ratings demonstrate the reasonably robust nature of self-reports as a measure of “state” and “trait” vividness (D’Angiulli, 2002; D’Angiulli and Reeves, 2002, 2007; D’Angiulli, 2009; Alter and Balcetis, 2010; Rabin et al., 2010; Pearson et al., 2011). This particular issue is critical given the recent resurgence of use of the VVIQ in cognitive neuroscience – especially in the realm of neuroimaging (Amedi et al., 2005; Palmiero et al., 2010).

In summary, we found that trial-by-trial vividness ratings predict incidental recall, and the relationship cannot be attributed to depth of elaboration or metacognitive processes related to self-appraisal of individual imagery ability, as measured by the VVIQ2. Our results suggest that vividness of imagery makes implicit information available to consciousness, and to some extent, is linked with the associative processes through which phenomenal availability translates into access of incidental episodic memories. Therefore, we conclude, in certain conditions conscious phenomenological experience associated with imagery does not have a trivial role as it can have a critical influence on recall performance.

## Conflict of Interest Statement

The authors declare that the research was conducted in the absence of any commercial or financial relationships that could be construed as a potential conflict of interest.

## Figures and Tables

**Figure A1:**
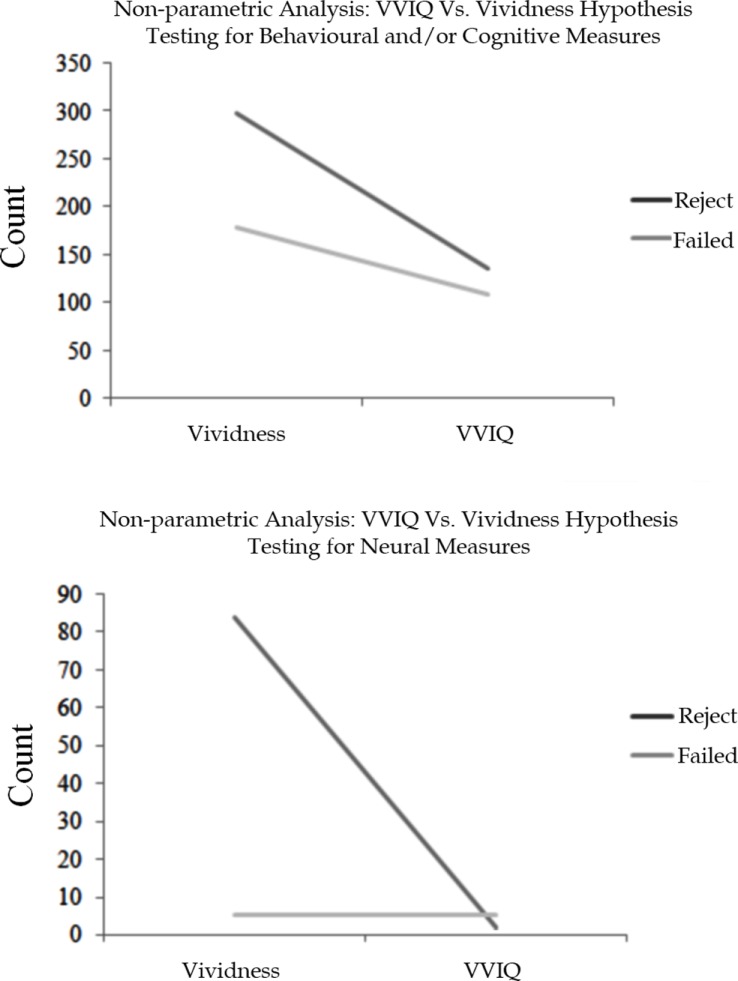
**(A)** Experimental outcomes for trial-by-trial vividness ratings and VVIQ with respect to behavioral and/or cognitive measures. **(B)** Experimental outcomes for trial-by-trial vividness ratings and VVIQ with respect to neural measures. The dark line refers to results which reject the null hypothesis, and the light line refers to results which fail to reject the null hypothesis.

**Figure A2:**
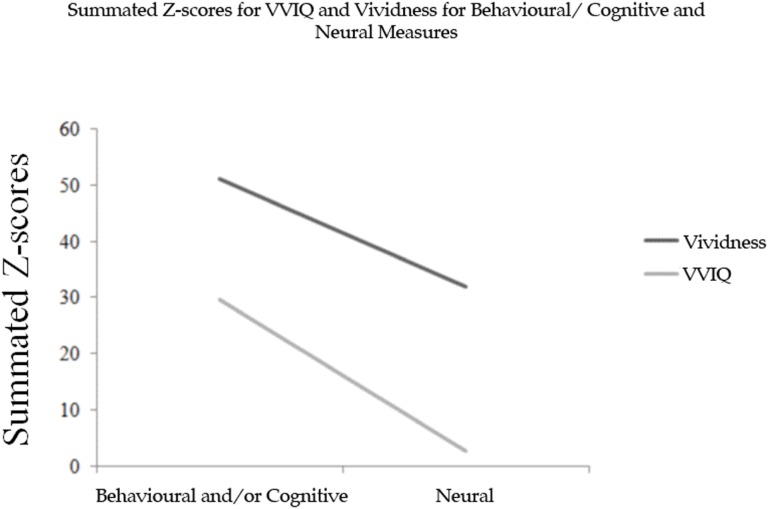
**Summed *Z*-scores for vividness and VVIQ for neural and behavioral and/or cognitive measures**.

## Appendices

### Appendix A

A linear mixed model was fit to the data to assess the contribution of the variables to linear change in vividness of imagery. The analysis was carried out using SPSS17. The variables in the model were evaluated by a Type III test. Since the sample size was not large, Restricted Iterative Generalized Least Square (RIGLS) estimation method was used. Table A1 shows the type III tests of fixed effects. (Cases with rating of “no image” (vividness rating value 1) were excluded from all analysis).

**Table A1:** **Type III tests of fixed effects in linear mixed model analysis testing the influences of stimuli and image generation time (RTs) on vividness ratings**.

Source	*df* numerator	*df* denominator	*F*	*P*
Intercept	1	632.643	3002.619	0.000
Stimuli	59	1000.202	1.588	0.004
RTs	1	1103.724	5.172	0.023
Stimuli × RTs	59	1046.513	0.956	0.572

*Dependent variable: vividness*.

To test how well the factors in the dataset predict recall, a logistic regression analysis was performed. The response for recall was recorded as 1 for recalled verbal descriptions and 0 for not recalled verbal descriptions. Explanatory variables included vividness, stimuli, and reaction time (RT). Table A2 displays model specifications, including the specified distribution and link function. Table A3 summarizes the results. Table A4 shows the full model results by predictor.

**Table A2:** **Basic repeated measure logistic regression model information**.

Dependent variable	Recall
Probability distribution	Binomial
Link function	Logit
Observation used	1490

**Table A3:** **Evaluation result for logistic regression predictive model for incidental recall using stimuli and vividness as predictor**.

		Predicted		
		Cues of imagined objects		Percentage correct
Observed		Non-recalled	Recalled	
Cues	Non-recalled	779	142	83.7
	Recalled	259	310	54.7
Overall percentage				72.2

**Table A4:** **Logistic regression analysis of vividness and incidental recall data**.

Predictor	β	SE	Wald	*df*	*p*	Exp (β)
*Vividness*			17.426	5	0.004	
Vividness (1)	−0.091	0.374	0.059	1	0.808	0.913
Vividness (2)	0.315	0.362	0.760	1	0.383	1.371
Vividness (3)	0.684	0.317	4.647	1	0.031	1.982
Vividness (4)	0.537	0.305	3.100	1	0.078	1.712
Vividness (5)	0.850	0.307	7.684	1	0.006	2.341
*Stimuli*			246.836	57	0.000	
Stimuli (1)	−2.776	0.751	13.658	1	0.000	0.062
Stimuli (2)	−2.511	0.690	13.256	1	0.000	0.081
Stimuli (3)	−2.485	0.690	12.955	1	0.000	0.083
Stimuli (4)	−0.873	0.659	1.755	1	0.185	0.418
Stimuli (5)	1.055	0.894	1.393	1	0.238	2.873
Stimuli (6)	−2.783	0.710	15.374	1	0.000	0.062
Stimuli (7)	−3.751	0.895	17.561	1	0.000	0.023
Stimuli (8)	−1.146	0.658	3.035	1	0.082	0.318
Stimuli (9)	−2.282	0.696	10.739	1	0.001	0.102
Stimuli (10)	−3.082	0.743	17.185	1	0.000	0.046
Stimuli (11)	−0.587	0.662	0.788	1	0.375	0.556
Stimuli (12)	−3.852	0.893	18.601	1	0.000	0.021
Stimuli (13)	−0.987	0.647	2.330	1	0.127	0.373
Stimuli (14)	−0.684	0.663	1.064	1	0.302	0.505
Stimuli (15)	−0.008	0.696	0.000	1	0.991	0.992
Stimuli (16)	−2.110	0.680	9.636	1	0.002	0.121
Stimuli (17)	−2.882	0.747	14.874	1	0.000	0.056
Stimuli (18)	−2.528	0.689	13.458	1	0.000	0.080
Stimuli (19)	0.254	0.744	0.117	1	0.733	1.289
Stimuli (20)	−2.955	0.745	15.724	1	0.000	0.052
Stimuli (21)	−3.342	0.797	17.586	1	0.000	0.035
Stimuli (22)	−1.610	0.647	6.186	1	0.013	0.200
Stimuli (23)	−2.172	0.679	10.230	1	0.001	0.114
stimuli(24)	−0.780	0.661	1.391	1	0.238	0.458
Stimuli (25)	−2.358	0.695	11.524	1	0.001	0.095
Stimuli (26)	−1.756	0.658	7.112	1	0.008	0.173
Stimuli (27)	−2.515	0.689	13.305	1	0.000	0.081
Stimuli (28)	−3.760	0.895	17.656	1	0.000	0.023
Stimuli (29)	−1.680	0.650	6.682	1	0.010	0.186
Stimuli (30)	−2.244	0.676	11.026	1	0.001	0.106
Stimuli (31)	−2.098	0.664	10.001	1	0.002	0.123
Stimuli (32)	−2.052	0.666	9.500	1	0.002	0.128
Stimuli (33)	−0.850	0.655	1.685	1	0.194	0.427
Stimuli (34)	−1.538	0.651	5.577	1	0.018	0.215
Stimuli (35)	−3.255	0.798	16.655	1	0.000	0.039
Stimuli (36)	−0.493	0.664	0.551	1	0.458	0.611
Stimuli (37)	−1.570	0.656	5.724	1	0.017	0.208
Stimuli (38)	−0.527	0.658	0.641	1	0.423	0.590
Stimuli (39)	−1.746	0.649	7.245	1	0.007	0.174
Stimuli (40)	−1.934	0.672	8.296	1	0.004	0.145
Stimuli (41)	−0.115	0.709	0.026	1	0.871	0.891
Stimuli (42)	0.262	0.723	0.132	1	0.716	1.300
Stimuli (43)	−0.490	0.667	0.539	1	0.463	0.613
Stimuli (44)	−0.764	0.651	1.376	1	0.241	0.466
Stimuli (45)	−2.352	0.674	12.196	1	0.000	0.095
Stimuli (46)	−1.479	0.645	5.259	1	0.022	0.228
Stimuli (47)	−3.562	0.901	15.633	1	0.000	0.028
Stimuli (48)	−2.927	0.747	15.370	1	0.000	0.054
Stimuli (49)	−1.717	0.650	6.989	1	0.008	0.180
Stimuli (50)	−1.421	0.643	4.883	1	0.027	0.241
Stimuli (51)	−2.051	0.665	9.501	1	0.002	0.129
Stimuli (52)	−1.712	0.682	6.299	1	0.012	0.181
Stimuli (53)	−0.028	0.691	0.002	1	0.967	0.972
Stimuli (54)	−2.172	0.679	10.238	1	0.001	0.114
Stimuli (55)	−2.407	0.691	12.132	1	0.000	0.090
Stimuli (56)	−1.138	0.735	2.395	1	0.122	0.321
Stimuli (57)	−2.455	0.691	12.612	1	0.000	0.086
*Constant*	0.664	0.576	1.329	1	0.249	1.943

*Variable(s) entered: vividness, stimuli*.

All 1490 valid observations were entered in the logistic regression model as the preliminary linear mixed modeling fitting analysis indicated that residual errors were only modestly correlated within each subject and were independent across subjects. Robustness to the violation of the assumption of independence was demonstrated by replicating the results with the following confirmatory repeated measure logistic regression model.

The dichotomous outcome for recall was further modeled with a repeated measure logistic regression analysis. The model was based on the probability of the largest value of response variable, which was 1. Two stimuli, which caused singularity of Hessian matrix, were removed from the dataset, resulting in 1441 observations (and no difference in the results). Models specifications, including the specified distribution and link function were same as the initial logistic regression model.

Type III test evaluated the effect of explanatory variables on recall accuracy in the purposed model. The test result showed that Vividness and stimuli were significant predictors for recall, χ^2^ = 14.77 and χ^2^ = 4276, *p* < 0.05 respectively (see Table A5). No other significance was found. Table A6 shows estimation for parameters in the model. Table A7 shows validity of predicted probabilities. The prediction for descriptions which were not recalled was more accurate than that for the verbal descriptions which were, 50.5% of non-recalled descriptions and 22% of recalled descriptions were correctly predicted. This confirmed that the model had overall 72.5% accuracy.

**Table A5:** **Type III test of model effects for repeated measure logistic regression model**.

Source	Wald χ^2^	*df*	*p*
(Intercept)	22.744	1	0.000
Vividness	14.766	5	0.011
RT	0.050	1	0.824
Vividness × RT	6.972	5	0.223
Stimuli	4276.081	25	0.000

*Dependent variable: recall*.

**Table A6:** **Parameter estimate for repeated measure logistic regression**.

			95% Wald confidence interval			Hypothesis test	
Parameter	β	SD	Lower	Upper	Wald	*df*	*P*
*(Intercept)*	0.905	0.4538	–	1.794	3.976	1	0.046
[vividness = 2.00]	1.136	0.3524	0.445	1.826	10.383	1	0.001
[vividness = 3.00]	0.748	0.3818	0.000	1.496	3.837	1	0.050
[vividness = 4.00]	0.608	0.4688	−0.311	1.526	1.680	1	0.195
[vividness = 5.00]	0.162	0.2989	−0.424	0.748	0.295	1	0.587
[vividness = 6.00]	0.356	0.2540	−0.142	0.854	1.965	1	0.161
[vividness = 7.00]	0^a^	–	–	–	–	–	–
*RT*	0.004	0.0054	−0.007	0.015	0.540	1	0.462
[vividness = 2.00] × s	−0.025	0.0188	−0.062	0.012	1.723	1	0.189
[vividness = 3.00] × s	0.018	0.0130	−0.008	0.043	1.892	1	0.169
[vividness = 4.00] × s	−0.005	0.0132	−0.031	0.021	0.147	1	0.701
[vividness = 5.00] × s	0.000	0.0079	−0.016	0.015	0.005	1	0.942
[vividness = 6.00] × s	−0.004	0.0100	−0.023	0.016	0.146	1	0.702
[vividness = 7.00] × s	0^a^	–	–	–	–	–	–
*Stimuli*							
[stimuli = 1.00]	−2.481	0.6790	−3.811	−1.150	13.345	1	0.000
[stimuli = 2.00]	0.293	0.8285	−1.331	1.917	0.125	1	0.723
[stimuli = 3.00]	0.036	0.7209	−1.377	1.449	0.002	1	0.960
[stimuli = 4.00]	0.023	0.5302	−1.016	1.062	0.002	1	0.965
[stimuli = 5.00]	−1.597	0.4804	−2.538	−0.655	11.046	1	0.001
[stimuli = 6.00]	−3.531	0.7974	−5.094	−1.968	19.609	1	0.000
[stimuli = 7.00]	0.318	0.6115	−0.880	1.517	0.271	1	0.603
[stimuli = 8.00]	1.291	0.9455	−0.563	3.144	1.863	1	0.172
[stimuli = 9.00]	−1.313	0.6087	−2.506	−0.120	4.650	1	0.031
[stimuli = 10.00]	−0.152	0.6943	−1.513	1.209	0.048	1	0.827
[stimuli = 11.00]	0.624	0.6256	−0.603	1.850	0.993	1	0.319
[stimuli = 12.00]	−1.936	0.5493	−3.013	−0.860	12.428	1	0.000
[stimuli = 13.00]	1.380	0.6474	0.111	2.649	4.542	1	0.033
[stimuli = 14.00]	−1.511	0.5988	−2.685	−0.337	6.368	1	0.012
[stimuli = 15.00]	−1.784	0.5815	−2.924	−0.644	9.410	1	0.002
[stimuli = 16.00]	−2.427	0.5536	−3.512	−1.342	19.224	1	0.000
[stimuli = 17.00]	−0.305	0.5736	−1.429	0.819	0.282	1	0.595
[stimuli = 18.00]	0.417	0.7336	−1.020	1.855	0.324	1	0.569
[stimuli = 19.00]	0.066	0.7188	−1.343	1.475	0.008	1	0.927
[stimuli = 20.00]	−2.730	0.7174	−4.136	−1.324	14.485	1	0.000
[stimuli = 21.00]	0.466	0.5001	−0.514	1.446	0.869	1	0.351
[stimuli = 22.00]	0.873	0.6202	−0.342	2.089	1.983	1	0.159
[stimuli = 23.00]	−0.877	0.6083	−2.069	0.315	2.079	1	0.149
[stimuli = 24.00]	−0.297	0.7287	−1.725	1.132	0.166	1	0.684
[stimuli = 25.00]	−1.656	0.6106	−2.853	−0.460	7.359	1	0.007
[stimuli = 26.00]	−0.097	0.7548	−1.576	1.382	0.016	1	0.898
[stimuli = 27.00]	−0.711	0.6595	−2.004	0.581	1.164	1	0.281
[stimuli = 28.00]	0.054	0.6819	−1.282	1.391	0.006	1	0.937
[stimuli = 29.00]	1.313	0.9485	−0.546	3.172	1.915	1	0.166
[stimuli = 30.00]	−0.786	0.6830	−2.125	0.552	1.326	1	0.249
[stimuli = 31.00]	−0.227	0.6441	−1.489	1.036	0.124	1	0.725
[stimuli = 32.00]	−0.366	0.6257	−1.592	0.860	0.342	1	0.559
[stimuli = 33.00]	−0.410	0.7467	−1.874	1.053	0.302	1	0.583
[stimuli = 34.00]	−1.615	0.6717	−2.931	−0.298	5.778	1	0.016
[stimuli = 36.00]	−0.931	0.6474	−2.200	0.338	2.069	1	0.150
[stimuli = 37.00]	0.881	0.7813	−0.650	2.412	1.272	1	0.259
[stimuli = 38.00]	−1.978	0.5974	−3.149	−0.807	10.958	1	0.001
[stimuli = 39.00]	−0.934	0.6869	−2.280	0.412	1.849	1	0.174
[stimuli = 40.00]	−1.942	0.5491	−3.018	−0.866	12.513	1	0.000
[stimuli = 41.00]	−0.726	0.5983	−1.898	0.447	1.471	1	0.225
[stimuli = 42.00]	−0.527	0.7072	−1.913	0.860	0.554	1	0.457
[stimuli = 43.00]	−2.346	0.6794	−3.678	−1.014	11.922	1	0.001
[stimuli = 44.00]	−2.760	0.6862	−4.104	−1.415	16.175	1	0.000
[stimuli = 45.00]	−1.974	0.6386	−3.226	−0.723	9.558	1	0.002
[stimuli = 46.00]	−1.686	0.6688	−2.997	−0.375	6.355	1	0.012
[stimuli = 48.00]	−0.120	0.5964	−1.289	1.049	0.041	1	0.840
[stimuli = 49.00]	−0.994	0.5766	−2.124	0.136	2.971	1	0.085
[stimuli = 50.00]	1.164	0.7937	−0.392	2.719	2.150	1	0.143
[stimuli = 51.00]	0.488	0.7849	−1.050	2.027	0.387	1	0.534
[stimuli = 52.00]	−0.759	0.6465	−2.026	0.508	1.379	1	0.240
[stimuli = 53.00]	−1.054	0.6764	−2.380	0.271	2.430	1	0.119
[stimuli = 54.00]	−0.415	0.5582	−1.509	0.679	0.553	1	0.457
[stimuli = 55.00]	−0.753	0.6128	−1.954	0.448	1.509	1	0.219
[stimuli = 56.00]	−2.425	0.7889	−3.971	−0.879	9.448	1	0.002
[stimuli = 57.00]	−0.292	0.6341	−1.535	0.950	0.213	1	0.645
[stimuli = 58.00]	−0.102	0.6570	−1.390	1.185	0.024	1	0.876
[stimuli = 59.00]	−1.387	0.7481	−2.853	0.079	3.439	1	0.064
[stimuli = 60.00]	0^a^	–	–	–	–	–	0.015

*Dependent variable: recall*.

*^a^Set to zero because this parameter is redundant*.

**Table A7:** **Predicted recall value from repeated measure logistic regression model**.

		Predicted category value		
Recalled		0.00	1.00	Total
0.0 (no)	Count	728	144	872
	% of Total	50.5	10.0	60.5
1.00 (yes)	Count	252	317	569
	% of Total	17.5	22.0	39.5
Total	Count	980	461	1441
	% of Total	68.0	32.0	100.0

### Appendix B

A corpus of 66 peer-reviewed experimental journal articles representing 4.32% of the literature available through PsycINFO, and containing the keyword “vividness” was randomly compiled by a research assistant naive to the purposes of the study (see Appendix references). Random selection of a relevant representative sample can be defended as a sound, reasonable meta-analytic tactic, provided the selected sources are analyzed according to a set of predefined, *a priori* criteria (Rosenthal, 1991). As a prerequisite for inclusivity, any statistical outcome directly pertaining to the measures VVIQ and trial-by-trial vividness ratings were to be utilized in the analysis, except those pertaining to *post hoc* comparisons.

The analysis consisted of two phases, a preliminary non-parametric analysis, and a secondary parametric analysis. Data for the preliminary analysis was obtained by partitioning individual experimental outcomes into two 2 × 2 contingency tables. Upon partitioning each experimental outcome as either a significant or non-significant experimental outcome, and as either a VVIQ or trial-by-trial vividness subjective report, each datum was further categorized as either a neural or behavioral/cognitive objective measure.

The same dataset from the preliminary non-parametric analysis was utilized in the secondary parametric analysis. However, each binomial outcome was transformed into an exact probability value. Analytic accuracy was maintained by calculating probabilities from reported test statistics and degrees of freedom. If required, raw data was statistically analyzed anew from means and variance. This rule was strictly adhered to unless otherwise unavoidable, in which case probability signifiers were rounded to the reported cut-off (i.e., *p* < 0.05 was approximated as 0.05); however, it should be noted that rounding was required six times over the course of 863 entries. The resultant entries were then categorized as either VVIQ or trial-by-trial vividness subjective report, and as either a neural or behavioral/cognitive objective measure. All values within each category were summated, and divided by the square root of the number of entries within each category.

A non-parametric analysis examining experimental outcome between VVIQ and trial-by-trial vividness ratings is presented in Figures A1A,B. The data in Figure A1A represent the number of significant versus non-significant experimental outcomes for VVIQ and trial-by-trial vividness ratings for behavioral/cognitive objective measures. The data in Figure A1B represent the number of significant versus non-significant experimental outcomes for VVIQ and trial-by-trial vividness ratings for neural objective measures. A higher proportion of successes accompany trial-by-trial vividness ratings for both behavioral/cognitive and neural objective measures. This relationship is especially true for studies underlying the neural origin of vividness.

The trends observed in the preliminary analysis prompted the use of a more sensitive statistical procedure. Because the directionality of each statistical outcome was not immediately apparent, and degrees of freedom often exceed one for *F*-tests and Chi-square tests of significance, standard meta-analytic methodology was decidedly insufficient for such purposes (Rosenthal, 1991). Under these circumstances, Stouffer’s method of adding *Z*’s provides a straightforward and reasonable estimate (Mosteller and Bush, 1954; Rosenthal, 1991). Upon determining exact probability values for each entry introduced, the values were transformed into their standard normal deviates. These values were summated, and divided by the square root of the number of entries within each category. Data for the parametric analysis is shown in Figure A2. These data show the summated *Z*-scores for VVIQ and trial-by-trial vividness ratings for behavioral/cognitive and neural objective measures.

As evidenced by Figure A2, two trends remain especially salient. Firstly, trial-by-trial vividness ratings are consistently greater for behavioral/cognitive and neural measures. Secondly, behavioral/cognitive measures yield significantly greater values than those which are neural. These results suggest that trial-by-trial vividness ratings are a more effective means by which to measure the subjective experience of mental imagery. Furthermore, Fisher’s *Z*-transformation for experimental outcomes concerning the correlation between VVIQ scores and trial-by-trial vividness ratings for 21 entries retrieved from six of the peer-reviewed journal articles showed an average *Z_r_* of 0.154, and variability in these values ranged from *r* = −0.27, to *r* = 0.64. Consistent with the results of experiment 2, these results support the contention that trial-by-trial vividness self-reports and VVIQ scores share some descriptive properties of visual imagery; however, trial-by-trial vividness ratings seem to be much more resolved.
